# Editorial: Immunology of allogeneic hematopoietic stem cell transplantation

**DOI:** 10.3389/frtra.2025.1567882

**Published:** 2025-02-19

**Authors:** Carmelo Gurnari, Remy Dulery, Raffaella Greco, Sara Piccinelli

**Affiliations:** ^1^ Department of Biomedicine and Prevention, University of Rome Tor Vergata, Rome, Italy; ^2^ Translational Hematology and Oncology Research Department, Taussig Cancer Center, Cleveland Clinic, Cleveland, OH, United States; ^3^ Department of Medical Oncology, Dana-Farber Cancer Institute and Harvard Medical School, Boston, MA, United States; ^4^ Department of Clinical Hematology and Cellular Therapy, Hospital Saint-Antoine, Assistance Publique—Hôpitaux de Paris, INSERM, UMRs 938, Centre de Recherche Saint Antoine (CRSA), Sorbonne University, Paris, France; ^5^ Unit of Hematology and Bone Marrow Transplantation, IRCCS San Raffaele Hospital, Vita-Salute San Raffaele University, Milan, Italy

**Keywords:** immunology, allogeneic hematopoietic cell transplant, CAR (chimeric antigen receptor) t cells, GvHD (graft-vs.-host disease), immunogenomics

**Editorial on the Research Topic**
Immunology of allogeneic hematopoietic stem cell transplantation

Allogeneic Hematopoietic Stem Cell Transplantation (HSCT) remains the only curative treatment for several high-risk hematologic malignancies (1). The role of the immune response in allogeneic HSCT involves both the eradication of the disease via the graft-vs.-leukemia (GvL) effect and the development of some of the major complications of the transplant procedure, such as graft rejection, graft-vs.-host disease (GvHD), and infections. Recently, there has been an increased interest in the field of cancer immunology and immunotherapy, which has also been reflected in the HSCT field. Numerous studies have focused on the understanding of the immune biology of HSCT in order to reduce adverse effects and enhance the anti-cancer efficacy. Moreover, innovative immunotherapeutic approaches such as bispecific antibodies, checkpoint inhibitors, and chimeric antigen receptor (CAR) T-cells are increasingly being combined with allogeneic HSCT to improve its therapeutic efficacy (2).

The articles published in the present Research Topic provide a glimpse of some of the critical aspects of immune biology of allogeneic HSCT and their implications in translational practice. These contributions range from retrospective cohort studies to exemplificative case reports that offer insights into managing peculiar and complex clinical scenarios.

Acute GvHD is one of the major toxicities of allogeneic HSCT. Research in this area is focused on the validation of reliable biomarkers for risk-adapted therapy (3). New therapeutic strategies aiming to avoid broad immunosuppression are under investigation (4). Sun et al. described the role of monocytes as potential biomarker for the prevention and treatment of acute GvHD, through a comparative analysis of single-cell RNA sequencing data on peripheral blood of patients with and without this complication. Monocytes showed a marked increase and activation on day 21 post-transplantation, before the onset of GvHD, which aligned with clinical cohort results obtained from routine blood examinations. Moreover, these monocytes were able to induce a significantly higher proliferation rate of T cells. Ideally, such an early GvHD biomarker could be useful to guide clinical management of GvHD. Another subset of immune cells, namely gamma delta (γδ) T cells, though a minor fraction of the human T cell repertoire, play a crucial role in anti-infectious and anti-tumor responses in allogeneic HSCT. In a prospective study of 49 pediatric allogeneic HSCT recipients, Müller et al. identified a protective role for γδ T cells, particularly the Vδ2 + subset, against acute GvHD and Epstein–Barr virus (EBV) infection. Multivariate analyses confirmed these findings, supporting further exploration of γδ T cells as prognostic markers and potential targets for adoptive T cell transfer strategies after HSCT.

With regard to GvHD prediction from an immunogenomic standpoint, a new metric to gauge the immunopeptidome diversity, called HLA evolutionary divergence (HED), previously explored in a variety of hematological conditions (5–7), is studied in acute lymphoblastic leukemia (ALL) patients undergoing haploidentical HSCT by Cao et al.. Both class I and II HED metrics were calculated in 225 patients with ALL. While no differences were found in terms of survival outcomes, multivariate analysis indicated that a high class II HED for donor-recipient was an independent risk factor for the development of severe acute GvHD (*P* = 0.007), and that recipients with high class I HED had a more than two-fold reduced risk of relapse (*P* = 0.028).

Three studies explore the potential benefits of using umbilical cord blood (UCB) products. Niu et al. reported the outcomes of adult and pediatric patients with severe steroid-refractory acute GvHD who were treated with intravenous infusions of human umbilical cord-derived mesenchymal stromal cells (UC-MSCs). The overall response rate at day 28 was 52.3%, without serious adverse events. Poor outcomes were observed for patients with acute lower gastrointestinal and liver GvHD.

Zeng et al. described how UCB regulatory T cells (Tregs), which play a key role in immune balance, work in synergy with Ruxolitinib in GvHD treatment. This combination effectively alleviates GvHD while preserving the beneficial GvL effect, as demonstrated in xenogeneic preclinical models. Graft failure (GF) and poor graft function (PGF) are potential complications in allogeneic HSCT, particularly in recipients with donor specific antibodies (DSA). UCBs, known for their high stem cell content and low immunogenicity, have been shown to promote immune tolerance when co-infused in haploidentical HSCT. In a retrospective, single-center, controlled study, Wang et al. demonstrated that co-infusion of third-party UCBs in patients with HLA antibodies improved engraftment and reduced the incidence of chronic GvHD.

Three case reports provide valuable insights into the management of challenging clinical scenarios. Zhu et al. described an unusual case of isolated spinal cord involvement with B-cell lymphoid proliferation 11 months post-HSCT, followed six months later by EBV-positive NK/T-cell lymphoma with subcutaneous involvement. This case underscores the importance of maintaining a high suspicion for post-transplant lymphoid proliferations in the context of neurological complications after HSCT and highlights the need for early diagnosis to manage this potentially life-threatening condition. Liu et al. reported a case of relapsed/refractory ALK + anaplastic large cell lymphoma successfully treated with crizotinib and brentuximab vedotin as bridging therapy, followed by autologous HSCT and sequential anti-CD30 CAR T-cell therapy. This innovative combination therapy model offers promising guidance for managing this rare subtype of T-cell non-Hodgkin lymphoma and informs future clinical trial strategies. Finally, Miao et al. described a patient with relapsed/refractory acute myeloid leukemia receiving donor-derived CLL-1 CAR T-cell therapy for bridging to allogeneic HSCT after achieving remission, showing the promising efficacy of cellular therapies in the realm of myeloid malignancies.

The studies and case reports presented in this Research Topic underscore the dynamic interplay between immune biology and clinical practice in allogeneic HSCT. Advances in biomarker discovery, cellular therapies, and immunogenomics are shaping personalized strategies to reduce complications, enhance the quality of life, and improve outcomes. Moreover, the integration of innovative immunotherapies highlights the potential to extend curative options to even the most challenging cases. Moving forward, collaborative research is essential to optimize the therapeutic potential of allogeneic HSCT while addressing its limitations. While the studies in this Research Topic provide valuable insights, they also point to the need for prospective trials to further validate findings and refine treatment strategies. By bridging translational science and clinical application, the field is poised to offer transformative solutions for patients with high-risk hematologic malignancies.
